# Clinical characteristics and risk factors of gastroesophageal reflux disease in Vietnamese patients with upper gastrointestinal symptoms undergoing esophagogastroduodenoscopy

**DOI:** 10.1002/jgh3.12536

**Published:** 2021-03-26

**Authors:** Duc T Quach, Quyen T T Pham, Truc L T Tran, Nhu T H Vu, Quang D Le, Doan T N Nguyen, Ngoc L B Dang, Huy M Le, Nhan Q Le

**Affiliations:** ^1^ Department of Internal Medicine University of Medicine and Pharmacy at Hochiminh City Ho Chi Minh Vietnam; ^2^ Department of Endoscopy University Medical Center Ho Chi Minh Vietnam; ^3^ Department of Gastroenterology Gia‐Dinh's People Hospital Ho Chi Minh Vietnam; ^4^ Department of Gastroenterology Cho‐Ray Hospital Ho Chi Minh Vietnam; ^5^ Department of Surgical Pathology University of Medicine and Pharmacy at Hochiminh City Ho Chi Minh Vietnam

**Keywords:** Barrett's esophagus, gastroesophageal reflux disease, *Helicobacter pylori*, reflux esophagitis, risk factors, Vietnam

## Abstract

**Background and Aim:**

The risk factors associated with the increase in prevalence of gastroesophageal reflux disease (GERD) are not consistent across countries and there have been few studies in Asia in the past 10 years. This study was conducted to assess the features and risk factors of GERD in Vietnamese patients.

**Methods:**

A cross‐sectional study was conducted on 1947 out‐patients ≥18 years of age who were presented with upper gastrointestinal symptoms and underwent esophagogastroduodenoscopy. Reflux esophagitis was graded according to the Los Angeles classification. Endoscopically suspected Barrett's esophagus (BE) was recorded according to the Prague C and M criteria and biopsy was taken for histologic examination.

**Results:**

There were 511 (26.2%) patients with GERD, 242 (47.4%) with nonerosive reflux disease, and 269 (52.6%) with reflux esophagitis and/or BE. Epigastric pain, regurgitation, and heartburn were the chief complaints in 36.8%, 27.0%, and 9.2% of patients, respectively. Most of the patients with mucosal injury had reflux esophagitis in mild grade and BE in the form of C0M ≤2 (99.6%, 231/232 and 97.8%, 46/47, respectively). In multivariate analysis, hiatal hernia, male gender, waist‐to‐hip ratio (independent from general obesity), and smoking were risk factors for GERD while *Helicobacter pylori* infection was negatively associated with GERD.

**Conclusions:**

The majority of GERD patients had none or mild mucosal injury. Typical reflux symptoms, however, may not be the chief complaints. Central obesity would be more important than general obesity as a risk factor, while *H. pylori* infection was a *“protective”* factor for GERD in Vietnamese patients.

## Graphical abstract

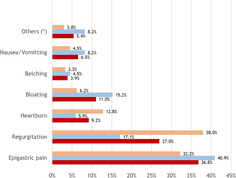

The majority of patients with gastroesophageal reflux disease (GERD) were with none or mild mucosal injury. Typical reflux symptoms, however, may not be the chief complaints. Central obesity would be more important than general obesity as a risk factor, while *Helicobacter pylori* infection was a “protective” factor for GERD in Vietnamese patients.

## Introduction

Gastroesophageal reflux disease (GERD) is prevalent all over the world.^1^, ^2^ Although there is evidence that the prevalence of reflux esophagitis has been increasing in the Asia Pacific region, there have been very few updated studies on this issue over the past 10 years.^3^, ^4^ There are several risk factors for GERD such as advanced age, gender, obesity, smoking, alcohol intake, and dietary behavior.^2^, ^3^, ^5^ And the key risk factors that would be associated with the increase in prevalence of GERD in Asia include obesity, consumption of a westernized diet, and the decreased prevalence of *Helicobacter pylori* (*H. pylori*) infection in the region.^3^ However, the risk factors for GERD are not consistent across countries.^2^, ^6^ Besides environmental factors, genetic factors may contribute to the differences across different ethnic groups.^3^ Vietnam is a rapidly growing country in Southeast Asia with a population of nearly 98 million, ranking as the 15th most populous country in the world.^7^ Surprisingly, there have been very few studies about GERD in Vietnamese patients. This study was conducted to assess the clinical features and risk factors for GERD in Vietnamese patients with upper gastrointestinal symptoms undergoing esophagogastroduodenoscopy. The results of this study would contribute to the local management strategy as well as the global understanding of the disease.

## Methods

### Subjects and settings

This is a cross‐sectional study conducted on out‐patients ≥18 years of age who were presented with upper gastrointestinal symptoms (e.g. nausea, vomiting, acid regurgitation, heartburn, epigastralgia, and abdominal fullness) and underwent esophagogastroduodenoscopy at the University Medical Center at Hochiminh City from August 2017 to March 2018. Patients were excluded if they were using anticoagulants or antiplatelets, diagnosed with esophageal cancer, esophageal varices, or active gastrointestinal bleeding, or had prior history of upper gastrointestinal surgery. Informed consent was obtained from all participants. The study protocol was approved by the Board of Ethics in Biomedical Research of University of Medicine and Pharmacy at Ho Chi Minh City (ID number: 271/DHYD‐HD, signed on 4th August 2017). A part of this data set has been published recently, in which the characteristics and risk factors of Barrett's esophagus (BE) were discussed.^8^

### Pre‐endoscopic assessment

Before patients underwent endoscopy, their demographic data were recorded. The typical reflux symptoms (i.e. heartburn and regurgitation) were carefully described to every patient. Waist and hip circumference were measured in every patient following instructions of the World Health Organization (WHO) Expert Consultation report.^9^ The body mass index (BMI) and waist‐to‐hip ratio (WHR) were calculated for all patients. All information was kept blinded to the endoscopists.

### Endoscopic assessment

Esophagogastroduodenoscopies were performed under topical anesthesia by five experienced endoscopists (VNTH, LQD, NDTN, DNLB, and LNQ) using Olympus scopes GIF‐160 or GIF‐Q180 (Olympus Co Ltd., Tokyo, Japan). Detailed information regarding their experience and training have been presented in detail in a recent paper of our group.^8^ Reflux esophagitis was graded according to the Los Angeles classification.^10^ Endoscopically suspected esophageal metaplasia, which was visible ≥1 cm above the gastroesophageal junction, was recorded according to the Prague C and M criteria; and at least one biopsy per 2 cm in tongues of endoscopic suspected BE, and four biopsies per 2 cm of circumferential suspected BE were taken for histologic examination.^11^

### Definitions

In this study, hiatus hernia was diagnosed if the separation between the squamocolumnar junction and the diaphragmatic impression was greater than 2 cm.^12^ Patients were considered to be affected by *H. pylori* infection when a local validated rapid urease test was positive within 1 h. The diagnostic criterion for BE was replacement of the normal distal squamous epithelial lining by columnar epithelium.^13^ Reflux disease with esophageal mucosal injury was defined as having reflux esophagitis, BE, esophageal stricture, or esophageal ulcer. Nonerosive reflux disease (NERD) was defined as having troublesome typical reflux symptoms, which occurred at least twice a week with no evidence of esophageal mucosal injury.^14^ GERD consists of NERD and reflux disease with esophageal mucosal injury.

### Statistical analysis

Continuous and categorical variables were expressed as mean ± standard deviation and 95% confidence interval (95% CI), respectively. Continuous variables were analyzed using Student *t* test or one‐way analysis of variance as appropriate. Categorical variables were analyzed using Pearson Chi‐squared test. Univariable and multivariable analyses using logistic regression were performed to identify the risk factors for GERD. All tests were two‐sided and performed at the 5% level of significance. All statistical calculations were performed with SPSS version 20.0 for Windows software (SPSS, Chicago, IL, USA).

## Results

### Demographic and endoscopic characteristics of the patients in the study

A total of 1947 patients were recruited. The male‐to‐female ratio was 1:1.2 and the mean age was 42.5 ± 12.0. The demographic and endoscopic characteristics of the patients in this study are given in Table 1. There were 511 (26.2%) patients with GERD, including 242 (47.4%) patients with NERD and 269 (52.6%) with reflux esophagitis and/or BE. Histologically, two out of 47 (4.2%) patients with BE had low‐grade dysplasia. The endoscopic characteristics of GERD with mucosal injury are given in Table 2.

**Table 1:** Demographic and endoscopic characteristics of the patients in the study

Characteristics^†^	Whole sample (*N* = 1947)	Gastroesophageal reflux disease (+) (*n* = 511)	Gastroesophageal reflux disease (−) (*n* = 1436)	*P* value
Male sex	890 (45.7)	292 (57.1)	598 (41.6)	<0.001
Age (years)	42.5 ± 12.0	43.2 ± 11.8	42.2 ± 12.1	0.125
Body mass index (kg/m^2^)	22.3 ± 3.0	22.5 ± 3.0	22.3 ± 3.0	0.092
Waist‐to‐hip ratio	0.84 ± 0.07	0.85 ± 0.07	0.84 ± 0.65	<0.001
Ex or current smoker	440 (22.6)	163 (31.9)	277 (19.3)	<0.001
Alcohol intake (≥1 drink/week)	565 (29.0)	191 (37.4)	374 (26.0)	<0.001
Hiatal hernia	44 (2.3)	25 (4.9)	19 (1.3)	<0.001
Gastric ulcer	37 (1.9)	7 (1.4)	30 (2.1)	0.306
Duodenal ulcer	40 (2.1)	11 (2.2)	29 (2.0)	0.855
Gastric cancer	5 (0.3)	1 (0.2)	4 (0.3)	0.751
*Helicobacter pylori* (+)	644 (33.1)	148 (29.0)	496 (34.5)	0.021

^†^ Data were presented as *n* (%) or mean ± SD.

**Table 2:** Characteristics of mucosal injury in patients with gastroesophageal reflux disease

Endoscopic characteristics of mucosal injury	*n/N* (%)
Reflux esophagitis	222/269 (82.5)
Barrett's esophagus	37/269 (13.8)
Barrett's esophagus and reflux esophagitis	10/269 (3.7)
The severity of reflux esophagitis (Los Angeles classification)	
Grade A	190/232 (81.9)
Grade B	41/232 (17.7)
Grade C	1/232 (0.4)
Grade D	0
Barrett's esophagus (Prague classification)	
C ≤ 1 M1	41/47 (87.2)
C ≤ 1 M2	5/47 (10.6)
C5M5	1/47 (2.2)

### Symptoms of patients with gastroesophageal reflux disease

**Figure 1:**
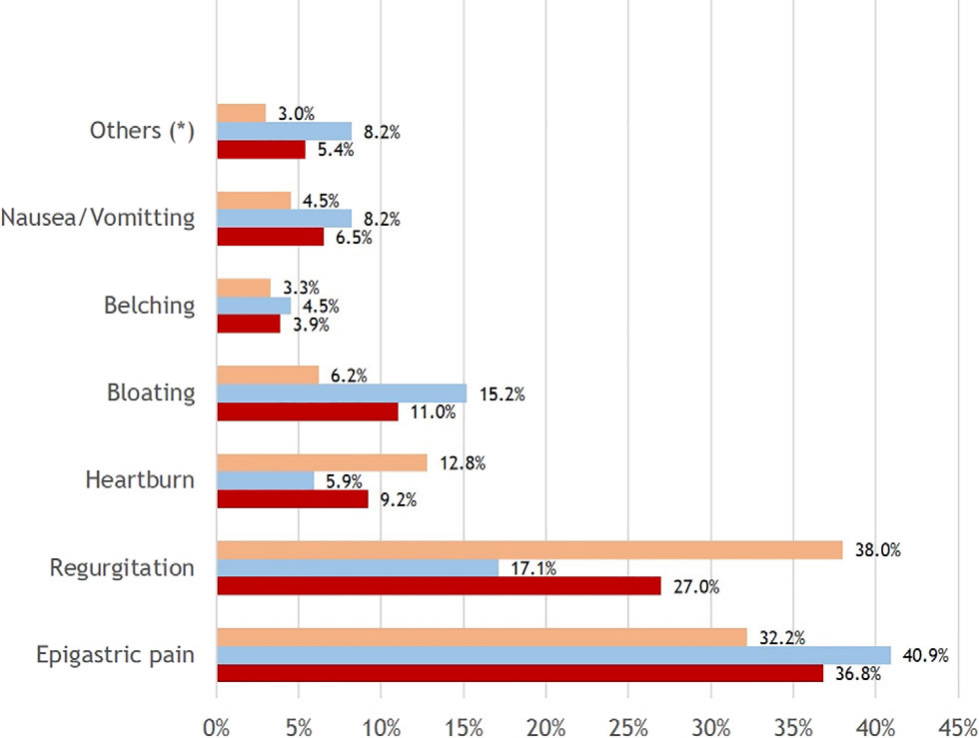
Chief complaints in patients with gastroesophageal reflux disease (GERD). NERD, nonerosive reflux disease; (*): Dysphagia, dry throat, or burning sensation in throat. 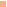, GERD with mucosal injury; 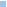, NERD; 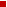, GERD total.

Typical reflux symptoms (i.e. heartburn and/or regurgitation) were present in 379 (74.2%) patients with GERD. However, there were only 137 out of 269 (50.9%) patients with mucosal injury had these symptoms. Regarding the chief complaints of patients with GERD, epigastric pain and regurgitation were the two most common symptoms. And only 47 (9.2%) patients were presented with heartburn as their chief complaint (Fig. 1).

### Factors associated with gastroesophageal reflux disease

In univariate analysis, there were significant associations between GERD and gender, BMI, WHR, smoking, alcohol intake, hiatal hernia, and *H. pylori* infection (Table 1). In multivariate analysis, the associations between GERD and BMI and alcohol intake were not significant. Hiatal hernia was the most predominant associated factor and *H. pylori* infection was negatively associated with GERD with the odds ratios (ORs) of 3.130 (95% CI, 1.689–5.800) and 0.777 (95% CI, 0.620–0.973), respectively (Table 3).

**Table 3:** Risk factors for gastroesophageal reflux disease on multivariate analysis

	Odds ratio	95% confidence interval	*P* value
Age	1.002	0.993–1.012	0.629
Male gender	1.366	1.010–1.847	0.043
Waist‐to‐hip ratio	1.362	1.086–1.708	0.007
Ex or current smoker	1.454	1.095–1.931	0.010
Alcohol intake (≥1 drink/week)	1.089	0.813–1.460	0.567
Hiatal hernia	3.130	1.689–5.800	0.000
*Helicobacter pylori* infection	0.777	0.620–0.973	0.028

## Discussion

This study showed that GERD was very popular among Vietnamese patients with upper gastrointestinal symptoms. However, typical reflux symptoms were not the chief complaints in a significant proportion of patients with GERD. This finding has also been reported in other Asian populations.^5^, ^6^, ^15^ The most widely accepted explanation is that most Asian languages do not have the equivalent of “heartburn”.^5^ Indeed, our local data in 2004 showed that the prevalence of typical reflux symptoms among Vietnamese patients with reflux esophagitis significantly increased from 20%, if patients were simply asked whether they had heartburn and regurgitation, to 63% if the interviewers spent time describing typical reflux symptoms.^16^ In this study, although we have paid attention to this point and spent time on explaining reflux symptoms to patients, epigastric pain was still the most common chief complaint among patients with GERD. This means that typical reflux symptoms were less likely to be considered by patients as an important issue. The explanation could be that the main concern of patients who sought consultation at hospital was gastric cancer, a common malignancy in Vietnam. Indeed, a previous study in Japan reported that patients rarely visited hospitals with reflux symptoms as their chief complaints, although these symptoms were prevalent in the primary care population.^17^

Regarding the clinical spectrum of GERD, our study showed that nearly half of the patients with GERD had esophageal mucosa injury. However, the majority of cases with reflux esophagitis in our study were in mild grade and most cases with BE were in the form of C0M ≤2 with no dysplasia. This finding is clinically important as it shows that the management strategy of GERD in Vietnam is mainly related to controlling the symptoms rather than dealing with serious complications.

Regarding the risk factors for GERD, our study found that the most predominant risk factor in Vietnamese patients was hiatal hernia. However, similar to other Asian studies, that its prevalence was very low suggests that this factor could not be the main reason for the increase of GERD prevalence over time. Smoking was also confirmed as a risk factor in our study. However, some other factors, which had been identified in other populations as risk factors for GERD such as advanced age and alcohol intake, were not significantly associated with GERD in our study.^2^, ^5^, ^6^ In contrast to previous studies showing a female predominance in GERD, we found that male gender was a risk factor for GERD in Vietnam. These differences may be due to the differences in diet, diet behavior, or genetic factors, which were not fully documented in our study and need to be further explored.

Overweight has been shown to be a major factor in the increase in incidence of GERD in Asia.^3^, ^5^, ^6^ Subjects who are overweight have significantly higher distal esophageal acid exposure.^18^, ^19^ BMI was strongly positively related to the frequency of reflux symptoms, and obese people were almost three times as likely to experience these symptoms as those of normal weight.^20^ However, central obesity could also cause partial hiatus herniation and short‐segment acid reflux.^21^ There is evidence showing that it could be more important than general obesity as a risk factor for reflux. The association between central obesity (independent of BMI) and reflux symptoms, however, was consistent in the White but not in the Black or Asian populations.^22^ Our study showed that central obesity was more important than BMI as a risk factor for GERD in Vietnamese patients. Indeed, a case–control study in Korea also showed that central obesity but not general obesity was an independent risk factor for NERD.^23^ There has been a significant increase in overweight rates observed for both genders and all age groups in Vietnam over time.^24^, ^25^ Consequently, this phenomenon would lead to a continuous increase in prevalence of GERD in the country.

There have been conflicting findings regarding the association between *H. pylori* infection and GERD in different ethnic groups, which may be due to the difference in gastric acid secretion status during the infection and genetic factors.^3^, ^5^ Previous studies showed that the majority of *H. pylori* strains isolated in Vietnam possessed *cagA* and gastric atrophy was popular among Vietnamese patients with the infection.^26^, ^27^ This could explain why our study found that Vietnamese patients with *H. pylori* infection had significantly lower risk for GERD (OR = 0.777, 95% CI, 0.620–0.973). Recent meta‐analyses consistently showed that eradication therapy for *H. pylori* increased the risk of reflux esophagitis.^28^, ^29^ As the prevalence of *H. pylori* infection in Vietnam has decreased significantly over the past 10 years due to massive eradication,^26^, ^27^ this could be another important factor contributing to the increase in prevalence of GERD in Vietnam in the future.

Our study has several limitations. First, it is a single hospital‐based study, which could not be reliably extrapolated to the general population in Vietnam. On the opposite, its strength was that it showed the majority of Vietnamese patients with GERD, even when recruited in a university hospital, were still with none or mild mucosal injury. Second, the study did not include criteria for investigating functional dyspepsia. The overlap between GERD and functional dyspepsia, which was probably common based on the high rates of epigastric pain and bloating among patients with GERD diagnosis, was not able to be evaluated and further study is required. Third, some possible risk factors related to diet, eating behavior, and associated drugs were not evaluated in this study.

In conclusion, GERD was prevalent in Vietnamese patients with upper gastrointestinal symptoms. The majority of patients were with none or mild mucosal injury. Typical reflux symptoms, however, may not be the chief complaints in more than half of the patients with GERD. WHR could be more important than BMI as a positive risk factor, while *H. pylori* infection is a “protective” factor for GERD in Vietnamese patients.
